# Brain Surgery

**Published:** 1895-09-21

**Authors:** 


### Progress in Surgery.

### BRAIN SURGERY.

At the German Congress of Surgery held at Berlin in April of this year, Professor Von Bergmann[1] selected as the subject of his address to the Congress "Recent Progress in Cerebral Surgery." While admitting that for obvious reasons the field of cerebral surgery will always be limited, he points out the enormous strides which have been made in this domain of our art, in consequence of our advanced knowledge of the localisation of cerebral function, and the perfection of our antiseptic methods. All our efforts, he says, must now be directed towards perfecting our means of diagnosis, and improving our surgical manipulations, chiefly in the direction of increasing the rapidity of brain operations. He advocates the use of a circular saw, worked by a small electric motor, for rapidly cutting out a large osseous flap from the skull, to enable the removal of large tumours. Such a method has been employed by Cotterill, of Edinburgh,[6] for some time.

**Head Injuries.**—Gross[2] contributes a valuable paper on "Scalping and Its Treatment." He refers to those cases in which the scalp is torn completely away from the head, usually as a result of the hair having been caught amongst the wheels or belts of machinery. The resulting wounds are exceedingly slow to heal on account (1) of the great loss of tissue, and (2) of the peculiar anatomical arrangement of parts in the wounded region. The separation takes place between the epicranial aponeurosis and the periosteum of the skull, and not infrequently the skin is peeled off for some distance beyond the limits of the skull proper, *e.g.*, from the face, neck or cheeks. The presence of dense bone under the granulations which spring from the periosteum hinders the natural contraction which accompanies cicatrisation, and the only aid to contraction lies in the elasticity of the surrounding parts. Spontaneous recovery is rare, and when it does occur is exceeding slow (Keeling four years, Syme eight years), while the risks from intercurrent affections are considerable. Skin grafting is our only resource in treating these cases. After discussing the relative merits of Reverdin, Thiersch, and animal grafting, and reporting fully a case treated by himself, the writer sums up that—(1) In these cases "the Ollier-Thiersch method of dermo-epidermic grafting is the surgeon's only resource for repairing the injury"; (2) that the grafts should be taken from the patient himself; (3) that special difficulties due to the anatomical features of the cranial region may necessitate supplementary grafting operations; and (4) that the results are decidedly favourable.

In a clinical lecture on a case of head injury, H. W. Page[2] describes the case of a cabman, who was thrown from his box and struck on the right temporal region, cheek, and orbit. There was no scalp wound nor external evidence of fracture, but the right eye was slightly proptosed, and the right pupil widely dilated and irresponsive to light. There was profound coma and stertorous breathing, with very slight rigidity of the left limbs and retraction of the right angle of the mouth. To exclude the possibility of the compression being due to an extravasation from the middle meningeal, and to give the patient his only chance, Mr. Page trephined over that artery, but with negative results. The patient died fifty-two hours later, with a temperature of 108 degrees Fahr. Post-mortem examination revealed a fracture involving the right orbital plate of the frontal. There were slight sub-arachnoid hæmorrhages on the surface of the occipital and temporal lobes. The interior of the ventricles was normal, but scattered through the right basal ganglia and internal capsule were various petechial hæmorrhages. Mr. Page attributes death to so-called concussion of the brain, and goes on to discuss the etiology and pathology of this condition. While the theory of contre-coup might explain the common surface lesions at the point struck, and at the opposite pole of the axis of percussion it fails to account for central lesions. He quotes the experiments of Miles, of Edinburgh, in support of Duret's opinion, that the serious disturbance of the cerebro-spinal fluid, set up by the blow on the skull, is the main factor in producing the lesions of concussion. Only on such a theory as that of Miles can he explain the post-mortem appearances in his own case.

In view of the interest at present taken in the effects of the Lee-Metford rifle bullets, Surgeon-Captain Porter's case[3] may be cited. A corporal was shot with this new magazine rifle, and the bullet took the following somewhat erratic course: Entering about three inches above the tip of the left internal condyle, it passed out again on the front of the arm a little higher up; one inch below the inner angle of the left eye, and passing through the nasal process of the superior maxilla was a small depressed wound. About the centre of the right half of the soft palate was a circular patch of ecchymosis; and lastly, behind the sterno-mastoid was a circular wound in the occipital bone. This was apparently the exit wound. The only untoward complication of the case was that the wad of the gun lodged in the arm wound, and there produced suppuration, otherwise the patient would have been discharged sixteen days after the infliction of the injuries. The man who fired the shot committed suicide before a description of the direction, &c., could be obtained from him.

**Operations in Head Injuries.**—Parker[4] comes to the following conclusions with regard to the advisability of operating in head injuries. (1) An exploratory incision should be made when there is any doubt as to the character of the injury; (2) punctured wounds must be enlarged for exploration and cleansing; (3) in all cases of depressed or fissured fracture operation is called for to elevate the depressed bone, or to explore the inner table; (4) large fragments, if not depressed, are to be left if surgically clean, even if denuded of dura and pericranium, to avert hernia-cerebri, vertigo, and other complications; (5) rigid asepsis, or antisepsis, is essential.

Bullard,[5] considering the same question, says that the primary indication for immediate operation is increased intra-cranial pressure. In the absence of paralysis and pupillary signs, the depth and progress of unconsciousness must be the guide. As a rule, operation is advisable when an adult patient cannot be roused by supra-orbital pressure, and when the pupils are irresponsive to light. Deepening unconsciousness and commencing paralysis indicate intracranial hæmorrhage, most likely from the middle meningeal artery. Large cranial openings, the incision of the dura, and the ligature of bleeding arteries are advocated.

Two new cases of successful operation for traumatic middle meningeal hæmorrhage are reported by Alexis Thomson[6] from the Royal Infirmary, Edinburgh. In one the symptoms of pressure on the motor areas did not appear till eight days after injury, and in this case no fracture existed. The other illustrates the value of antiseptic measures in compound fractures of the skull. In this case the skull was opened by means of the circular saw worked by a dental engine as suggested by Cotterill.

Tubby[7] reports a case of a gentleman who sustained a severe head injury by being thrown from his horse. A large piece of bone was broken off, and pressed into the great longitudinal fissure so as to separate the two hemispheres. Although able to walk home, compression symptoms ensued, and he was operated on thirty-four days after injury, and made a good recovery.

A most curious result of a cranial fracture is described and illustrated by Willson.[8] A man received extensive injuries to his frontal region from the bursting of a shell in the Chinese War of 1860. On his recovery he returned to England, where he recently died of acute bronchitis. The whole frontal region from the supra-orbital eminences to the coronal suture was markedly depressed, forming a deeply concave surface, across which there passed a transverse bar of bone two and a-half inches long by one-third of an inch deep, completely invested in skin. Between the bar and the skull a No. 12 catheter could be freely passed.

[1] Med. Week, Ap. 26, 1895. [2] Med. Week, May 24, 1895. [3] B.M.J., Feb. 16, 1895. [4] Jour. Amer. Med. Assoc., Feb., 1895. [5] Boston Med. and Surg. Journ., 1895. [6] Edin. Med. Journ., Jan., 1895 [7] B.M.J., Ap. 13, 1895. [8] B.M.J., June 1, 1895.

